# Integrated gross and microanatomical analysis of the periportal lymphatic system in human liver

**DOI:** 10.1002/jhbp.12127

**Published:** 2025-03-04

**Authors:** Yuto Mitsuhashi, Hiroshi Shimoda, Kotaro Umemura, Takuji Kagiya, Kentaro Sato, Hirokazu Narita, Tomohiro Chiba, Keinosuke Ishido, Norihisa Kimura, Kenichi Hakamada

**Affiliations:** ^1^ Department of Gastroenterological Surgery Hirosaki University Graduate School of Medicine Hirosaki Aomori Japan; ^2^ Department of Anatomical Science Hirosaki University Graduate School of Medicine Hirosaki Aomori Japan; ^3^ Biomedical Science and Engineering Research Center Hakodate Medical Association Nursing and Rehabilitation Academy Hakodate Hokkaido Japan

**Keywords:** cadaver, liver, lymphatic anatomy, lymphatic dynamics, periportal lymphatic system

## Abstract

**Background:**

Although animal studies have indicated that the periportal lymphatic system is an important intrahepatic lymphatic route, detailed human studies are scarce. We studied the lymphatic pathways and dynamics around Glisson's capsule in human livers.

**Methods:**

We performed whole‐mount immunostaining for the liver using podoplanin and CD‐31 to elucidate the lymphatic vessel distribution around the hepatic triad (HT). Next, 5 mL of India ink was injected into the liver parenchyma to observe the lymph fluid dynamics around Glisson's capsule. Lastly, immunohistochemical staining for CCL21, a chemokine important for lymphocyte migration, and its receptor CCR7 was performed to observe the lymphocyte dynamics within Glisson's capsule.

**Results:**

Lymphatic vessels with blind‐ended structures were observed around the peripheral HT. These vessels ran longitudinally, forming a network with numerous blind ends around HT. Ink distribution was observed within gaps and capillary lymphatic vessels in Glisson's capsule. These gaps were CCL21‐positive, and a concentration gradient was observed toward the capillary lymphatic vessels.

**Conclusions:**

The intrahepatic lymphatic fluid enters the capillary lymphatic vessels via the extravascular pathway, and lymphocyte components enter the capillary lymphatic vessels through the CCR7/CCL21 transport system in the extravascular pathway. This is the first study to elucidate the human intrahepatic periportal lymphatic system.

## Graphical abstract

Using whole‐mount immunostaining, Mitsuhashi and colleagues conducted detailed visualization of lymphatic pathways surrounding the hepatic Glisson's capsule in the human liver. Distinct lymphatic structures and lymphocyte transport via CCR7/CCL21 signaling along the extravascular pathway were identified. This is the first study to elucidate the intrahepatic periportal lymphatic system in humans.

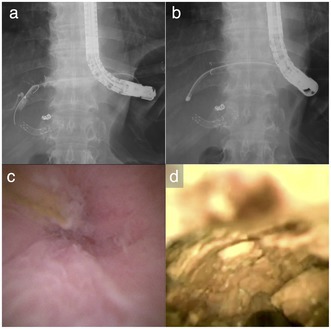

## 1 INTRODUCTION

Studies of macroscopic anatomy have shown that malignant hepatic tumors metastasize to extrahepatic sites through lymphatic pathways as the cancer progresses.^1^ Hepatocellular carcinoma and intrahepatic cholangiocarcinoma are primary malignant liver tumors. They metastasize lymphatically from the hepatic hilum toward the abdominal aorta. This metastasis is associated with an extremely poor prognosis.^2^, ^3^ However, detailed studies on intrahepatic lymphatic drainage are limited, and elucidating the intrahepatic lymphatic pathways is vital for overcoming the spread of these hepatic malignancies.

Intrahepatic lymphatic fluid flows out of the liver through three pathways: the periportal lymphatic, hepatic venous lymphatic, and superficial lymphatic systems.^4^, ^5^ Among these, >80% of intrahepatic lymphatic fluid drains into the capillary lymphatic vessels within Glisson's capsule, establishing the periportal lymphatic system as the primary pathway for hepatic lymph flow.^6^, ^7^, ^8^ The intrahepatic periportal lymphatic system has been mainly studied as a microscopic anatomy in experimental animals.^9^, ^10^ Ohtani et al.^10^ demonstrated that a tracer injected intravenously flows from the hepatic sinusoids into the Glisson's capsule in rats through an extravascular fluid pathway, a third pathway distinct from blood and lymphatic vessels. Other experimental animal studies have shown that hepatic lymph fluid in the periportal lymphatic system is filtered into Disse's space, a gap between hepatocytes and sinusoidal endothelial cells, and is then drained into the capillary lymphatic vessels within the Glisson's capsule through Mall's space,^11^ a gap between the liver's limiting plate and Glisson's capsule.^5^, ^10^ However, no such studies have been conducted on the intrahepatic periportal lymphatic system in humans.

Recently, we clarified the existence and relationship between the hepatic venous lymphatic system and intrahepatic lymph dynamics using human cadavers.^12^ Through en bloc excision of the liver from human cadavers and whole‐mount staining of intrahepatic lymphatic vessels, a lymphatic network was identified around the hepatic veins. This network could act as an outflow pathway for intrahepatic lymphatic fluid. Our previous study^12^ revealed that the lymphatic vessels around the hepatic veins originate from the peripheral portion of the hepatic veins, becoming denser and more continuous toward the proximal portion and main trunk. Furthermore, it was shown that intrahepatic lymphatic fluid flows out from the stroma of the external walls of the central and sublobular veins within the hepatic lobules into the lymphatic vessels in the peripheral portion. Thus, we elucidated the existence and function of the hepatic venous lymphatic system in the human liver. However, the study did not elucidate the details of the periportal lymphatic system. Therefore, this study aimed to microscopically elucidate the morphology of the intrahepatic periportal lymphatic system using our method. Furthermore, this study focused on the existence of the intrahepatic periportal lymphatic system and on the lymph dynamics, specifically examining the lymphatic fluid and lymphocyte components.

## 2 METHODS

### 2.1 Study subjects

This study used postmortem human liver specimens collected from three donors (Table 1). The donors were registered with Hirosaki University Shiragikukai, an organization for individuals who consent to donate their bodies posthumously, with agreement from both the individuals and their relatives. All donors were screened to confirm the absence of human immunodeficiency virus, hepatitis B virus, and hepatitis C virus infections. Additionally, postmortem consent from relatives was obtained. The cadavers were injected with 10% formalin and 5% phenol via the femoral artery to ensure proper fixation. None of the donors exhibited hepatic tumors or abdominal malformations. The Human Research Ethics Committee of Hirosaki University Graduate School of Medicine approved this study (Reference No. 2015‐226). Opt‐out consent was obtained from the relatives.

**TABLE 1:** Details of the three cadavers.

No.	Age	Sex	Cause of death	Preservation time until specimen collection	Pathological findings of the liver	Goal of the experiment
1	91	Female	Acute pneumonia	22 months	Almost normal	Three‐dimensional structure of lymphatic vessels around the hepatic triad
2	88	Female	Cerebral hemorrhage	15 months	Moderate hepatitis	Lymphatic dynamics around the hepatic triad
3	75	Male	Suffocation	22 months	Mild hepatitis	Lymphocyte dynamics around the hepatic triad

### 2.2 Whole‐mount en bloc immunohistochemistry for human liver specimens

After en bloc removal of the liver from the cadavers, the parenchyma was removed to expose the hepatic triad (HT) for whole‐mount immunostaining and stereomicroscopic examination. Under a surgical microscope, the hepatic parenchyma was manually removed to avoid damaging the liver vasculature. After rinsing the whole‐mount preparations in phosphate‐buffered saline (PBS) with 0.3% Triton X‐100 (FUJIFILM Wako Pure Chemical Corporation, Osaka, Japan), they were immersed in 0.3% H_2_O_2_ in PBS containing 0.1% sodium azide (FUJI FILM Wako Chemical Industries, Osaka, Japan) at room temperature for 30 min. The samples were then incubated in 5% normal goat serum (NGS; Vector Laboratories) at 4°C for 24 h, followed by a 7‐day incubation at 4°C with a mixture of podoplanin antibody (D2‐40, DAKO, Santa Clara, California; 1:100) and CD31 antibody (EP3095, Abcam, Cambridge, UK). This was followed by a 5‐day incubation in a mixture of alkaline phosphatase (AP)‐conjugated anti‐mouse goat IgG (Histofine Simple Stain MAX‐AP, Nichirei Biosciences, Tokyo, Japan) and peroxidase (PO)‐conjugated anti‐rabbit goat IgG (Histofine Simple Stain MAX‐PO, Nichirei Bioscience) at 4°C. Immunoreactions were visualized using VECTOR Blue (SK‐5300, Vector Laboratories, Burlingame, California) for AP and diaminobenzidine (DAB; Dojindo, Kumamoto, Japan) for PO and observed under an MVX10 dissection microscope with a DP80 digital imaging system (Olympus).

### 2.3 Ink injection

To study the pathways of intrahepatic lymphatic fluid, the liver was removed from the cadavers without damaging the inferior vena cava, portal vein, and hepatoduodenal ligament. Next, 5 mL of filtered ink (Kuretake, Nara, Japan) was manually injected into the parenchyma of the right hepatic lobe using a 21G needle. To fix the tissues, the liver was then immersed in Sol Mix (64% ethanol, 10% methanol, and 0.8% formalin) (Nihonkasei, Sendai, Japan) for 24 h. Subsequently, the liver was coronally sectioned at 1‐cm intervals to create tissue blocks of 2 × 1 cm. After paraffin embedding, 5‐μm‐thick sections were prepared for immunohistochemical staining and were observed under an optical microscope to examine the distribution of lymphatic vessels and ink around the intrahepatic Glisson's capsule.

### 2.4 Immunohistochemical staining and optical microscopy

For hematoxylin and eosin (HE) staining and immunohistochemistry, 5‐μm‐thick sections were prepared. Sequential sections from HE‐stained samples were deparaffinized and subjected to antigen retrieval by immersion in 0.01 M citrate buffer (pH 6.0) at 121°C for 15 min. Endogenous peroxidase activity was blocked by immersing sections in PBS (1/15 M, pH 7.4) containing 0.3% H_2_O_2_ and 0.1% sodium azide at room temperature for 20 min. The sections were incubated with 5% NGS for 15 min, followed by overnight incubation at 4°C with a mixture of rabbit anti‐human CD31 (Abcam) and mouse anti‐podoplanin (D2‐40, DAKO) antibodies. Similarly, sequential sections were treated with a mixture of rabbit anti‐CCR7/CKR7 (E271, Abcam) and mouse anti‐CCL21/6Ckine (Mo146‐2H26, Novus Biologicals, CO, USA) antibodies and a mixture of rabbit anti‐collagen III (ab7778, Abcam) and mouse anti‐collagen I (3G3, Abcam) antibodies. Sections were then reacted with mixtures of AP‐conjugated anti‐rabbit or anti‐mouse goat IgG and peroxidase (PO)‐conjugated anti‐rabbit or anti‐mouse goat IgG for 1 h at room temperature. Immunoreactive sites were visualized using VECTOR Blue for AP and DAB or PermaRed/HRP (K075, Diagnostic BioSystems, Pleasanton, CA, USA) for PO. Observations were made using a BX‐60 optical microscope equipped with a DP72 digital imaging system (Olympus, Tokyo, Japan).

### 2.5 Control immunostaining

For control immunostaining, antibodies preadsorbed with excess antigen (50–100 μg/mL diluted antibody; CD31, CCR7/CKR7, collagen III, smooth muscle actin, collagen I: Abcam; CCL21/6Ckine: Novus Biologicals) or nonimmune serum were used instead of the primary antibody. The staining results were confirmed to be completely negative.

## 3 RESULTS

### 3.1 Distribution of lymphatic vessels around the hepatic triad in humans

**FIGURE 1:**
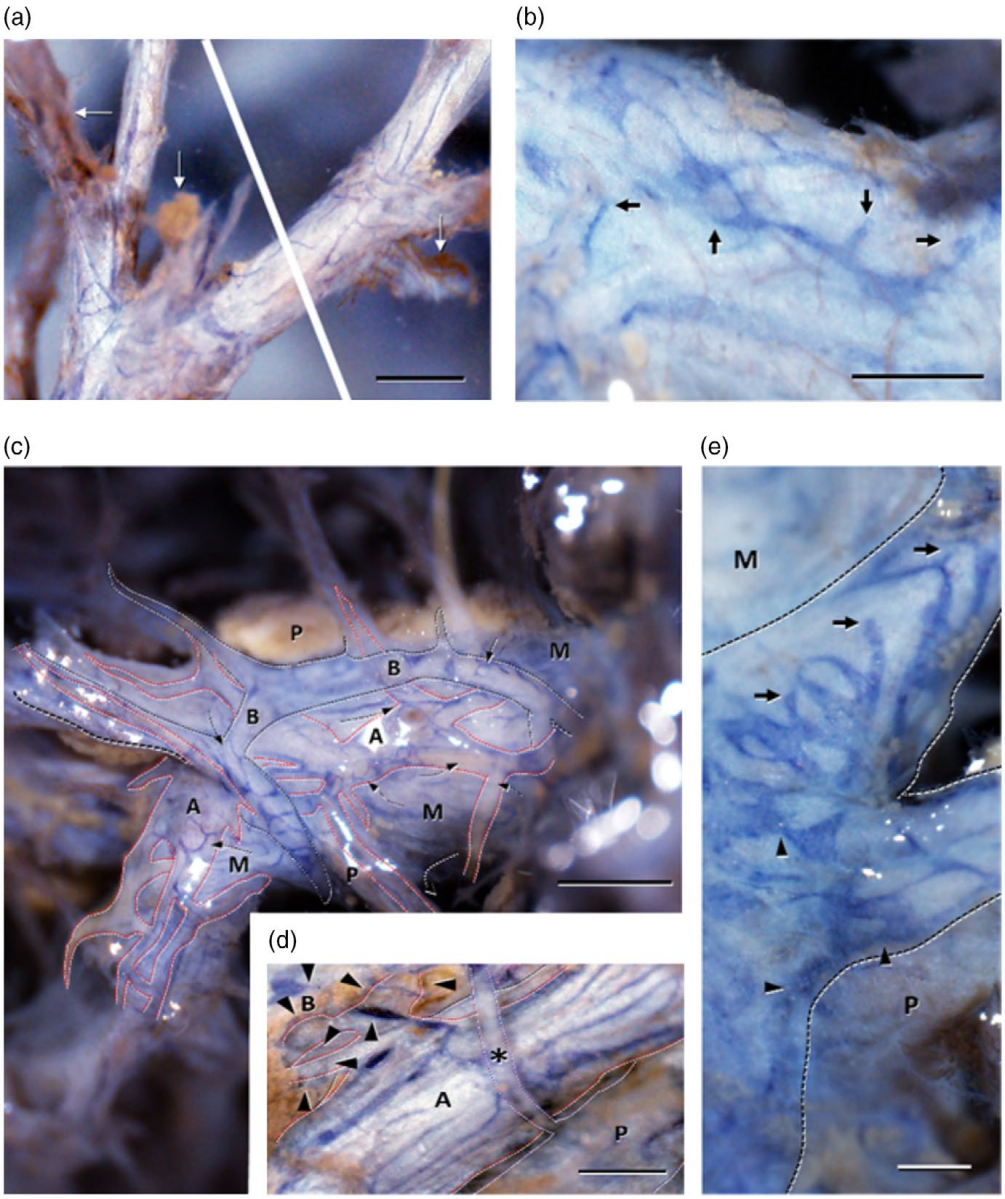
Stereomicroscopic images of whole‐mount preparations of the human intrahepatic portal tract immunostained for podoplanin and CD31. (a) The terminal portions of the portal tract show several slender initial lymphatic vessels (blue) with podoplanin immunoreactivity. Arrows indicate the residual sinusoids with CD31 immunoreactivity. (b) Higher magnification image of the terminal portion of the portal tract. The podoplanin‐immunopositive initial lymphatic vessels (blue) equipped with blind endings (arrows) are shown with the CD31‐immunopositive blood vessels (brown) on the surface of the portal tract. (c) An overview of the secondary segmental portal tract. The distribution of lymphatic capillaries (blue) is shown with the proper structure of the portal triad. Black and red broken lines indicate the contours of the interlobular bile ducts (B) and arteries (A), respectively. Broken arrows indicate the course of the lymphatics from Mall's space (M) to the interlobular artery (A) or bile duct (B). Lymphatic capillaries from the Mall's space (M) gradually converge on the segments of the interlobular artery and bile duct rather than on the interlobular vein (P). (d) Higher magnification image of a part of the secondary segmental portal tract shown in (c). Black and red broken lines indicate the contours of the interlobular bile ducts (B) and arteries (A), respectively. The interlobular artery contains many lymphatic capillaries (blue) running longitudinally. Arrowheads indicate thin lymphatic capillaries running along the arterial branch composing the peribiliary arterial plexus. The interlobular vein (P) shows a poor distribution of lymphatics compared with the interlobular bile duct (B) and artery (A). Asterisks indicate branches of the interlobular vein. (e) Higher magnification image of a part of the secondary segmental portal tract shown in (c). The interlobular bile duct shows a delicate meshwork of lymphatic capillaries (blue) with blind endings (arrows) and notches (arrowheads). M: Mall's space; P: Interlobular vein. Bars: 0.5 mm (a and b), 0.25 mm (c), 1 mm (d), and 0.5 mm (e).

Podoplanin‐positive lymphatic vessels and CD31‐positive blood vessels were observed within the HT, where the interlobular connective tissue and HT structure remained intact (Figure 1). Thin lymphatic vessels running along the HT were observed in the peripheral HT (Figure 1a). These lymphatic vessels had blind‐ended structures at the periphery. Furthermore, numerous lymphatic vessels with blind‐ended structures that joined the longitudinally running lymphatic vessels were observed (Figure 1b). In the central HT, the peripheral lymphatic vessels were noted to be condensed and increased in number. Furthermore, characteristic lymphatic structures were observed around the bile ducts and arteries (Figure 1c). The lymphatic vessels around the interlobular arteries ran longitudinally around the arteries (Figure 1d). Conversely, the lymphatic vessels were distributed sparsely around the interlobular portal veins (Figure 1d,e). While lymphatic vessels around the interlobular bile ducts were distributed along the arterial branches, forming the peribiliary vascular plexus (Figure 1d). These lymphatic vessels around the interlobular bile ducts formed a tubular, labyrinthine network with numerous blind ends at their tips (Figure 1e).

### 3.2 Distribution and dynamics of lymphatic fluid components in the human hepatic Glisson's capsule

**FIGURE 2:**
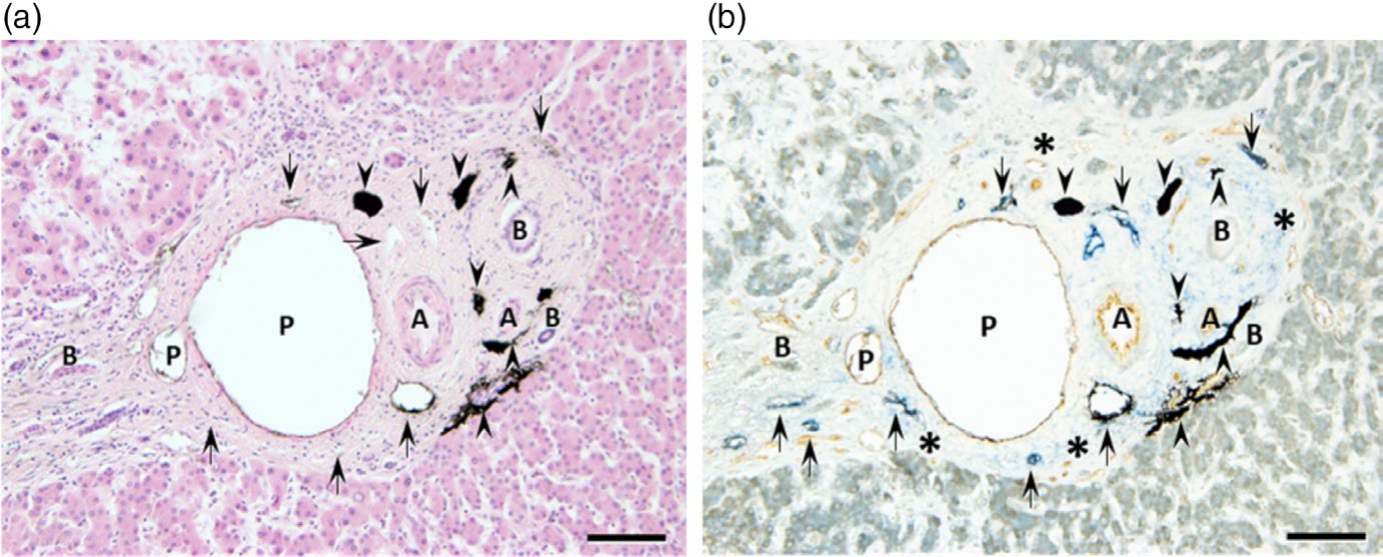
Microscopic image after ink injection into the liver parenchyma. Light micrographs of hematoxylin and eosin staining (a) and light microscopic images of immunostaining for podoplanin (blue) and CD31 (brown) (b) on tissue sections of the human liver. India ink (black, arrowheads) injected into the hepatic parenchyma before tissue collection was observed in Mall's space (asterisks) and the lymphatic vessels (arrows) immunopositive for podoplanin in Glisson's capsule. A, interlobular artery; B, interlobular bile duct; P, interlobular vein. Bars: 100 μm.

The liver tissues preinjected with ink were used to elucidate the distribution and dynamics of lymphatic vessels and fluid components in the human hepatic Glisson's capsule and surrounding tissues. The ink was found within the podoplanin‐positive capillary lymphatic vessels in Glisson's capsule (Figure 2a,b). Additionally, the capillary lymphatic vessels were extensively distributed between Mall's space and the HT, along with interlobular arteries and bile ducts, suggesting that fluid components move through the extravascular fluid pathway connecting these structures (Figure 2a,b).

### 3.3 Dynamics of lymphocytes in the human hepatic Glisson's capsule

**FIGURE 3:**
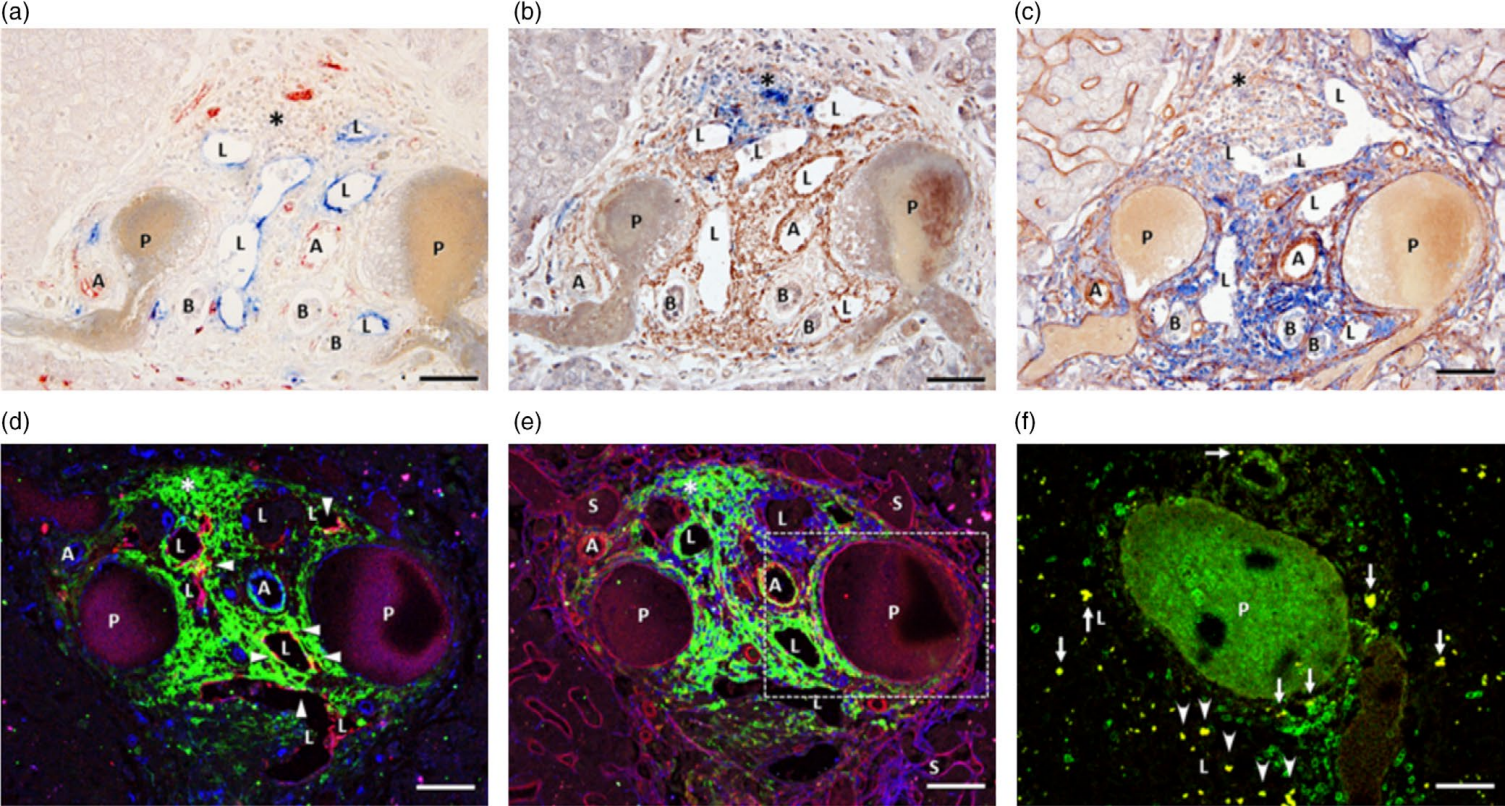
Multiple immunostainings for Glisson in the hepatic lobule. Optical fluorescence microscopic images indicated positive immunostaining for podoplanin (blue in a) and CD31 (red in a); for CCR7 (blue in b) and CCL21 (brown in b); for collagen I (blue in c) and collagen III (brown in c); for CCL21 (green in d), podoplanin (red in d), and CD31 (blue in d); for CCL21 (green in e), collagen III (red in e), and collagen I (blue in e); and for CD3 (green in f) and CCR7 (red in f) on serial tissue sections of the human liver. (a) Podoplanin‐immunopositive lymphatic vessels (blue, L) are distinguishable from CD31‐immunopositive blood vessels (red), including arteries (A) and capillaries. The interlobular vein (P) is faintly immunopositive for CD31, whereas interlobular bile ducts (B) are negative for both antigens. (b) Immunoreaction products for CCL21 (brown) are densely distributed around the lymphatics (L) and less in the following area of Mall's space, whereas CCR7‐immunopositive cells (blue) are observed in the CCL21‐immunopositive area between Mall's space and lymphatics. (c) The collagen III‐immunopositive tissue (brown) extends from the lymphatic vessels (L) to Mall's space or the HT, and most residual portal areas are filled with the collagen I‐immunopositive tissue (blue). (d) The reaction products for CCL21 (green) are observed on the podoplanin (red)‐immunopositive lymphatic endothelium (arrowheads, yellow) and around the lymphatic vessels (L) and in Mall's space. (e) The immunoreaction products for CCL21 (green) are exclusively distributed in the collagen III‐immunopositive tissues (red) but not in the collagen I‐immunopositive tissues (blue). (f) Higher magnification of an adjacent sectional image of the boxed area shown in (e). Some CD3 (green)‐immunopositive lymphocytes showing CCR7 (red) immunoreactivity (yellow) are seen around (arrows) and within (arrowheads) the lymphatic vessels (L). A, interlobular artery; B, interlobular bile duct; P, interlobular vein. Bars: 50 μm.

Immunohistochemical staining for CCR7, a chemokine receptor crucial for lymphocyte migration, and for its ligand CCL21, was used to elucidate lymphocyte dynamics in Glisson's capsule. The CCL21‐stained area was concentrated mainly around the capillary lymphatic vessels in the hepatic Glisson's capsule, forming a density gradient toward Mall's space (Figure 3a,b). Additionally, CCR7‐positive cells were abundantly distributed in the CCL21‐positive areas between Mall's space and the lymphatic vessels, with some cells infiltrating the lymphatic vessels (Figure 3b). Sequential sections were immunohistochemically stained for collagen types I and III. The CCL21‐stained areas shown in Figure 3b corresponded to the areas stained for collagen type III, a component of reticular fibers (Figure 3c). Furthermore, triple immunofluorescence staining for podoplanin (lymphatic vessels, red), CD31 (blood vessels, blue), and CCL21 (green) in the hepatic Glisson's capsule revealed that CCL21‐positive areas were more densely distributed around the capillary lymphatic vessels (Figure 3d). Additionally, triple immunofluorescence staining for collagen type I (blue), collagen type III (red), and CCL21 (green) showed that collagen type III‐positive areas overlapped with CCL21‐stained regions, whereas collagen type I was predominantly observed around blood vessels. These results indicate that CCL21‐positive areas coincide with collagen type III‐stained regions but are absent in collagen type I distribution areas, such as around the portal veins (Figure 3e). Furthermore, to confirm that the CCR7‐positive cells in the hepatic Glisson's capsule are lymphocytes, double immunostaining with anti‐CD3 antibodies was performed. The results showed that the CD3‐positive cells also reacted positively for CCR7, which appeared yellow, confirming that the CCR7‐positive cells in the hepatic Glisson's capsule were lymphocytes (Figure 3f). Furthermore, it was also shown that Glisson's capsule in the liver is rich in reticular fibers mainly comprising type III collagen, with abundant CCL21 expression. Moreover, it strongly suggests that these reticular fibers function as ligands for CCR7 expressed in lymphocytes. Therefore, it is suggested that lymphocytes pass through the reticular fibers via CCR7/CCL21 signaling and invade the capillary lymphatic vessels in Glisson's capsule, indicating that the reticular fibers act as an extravascular fluid pathway for lymphocytes.

**FIGURE 4:**
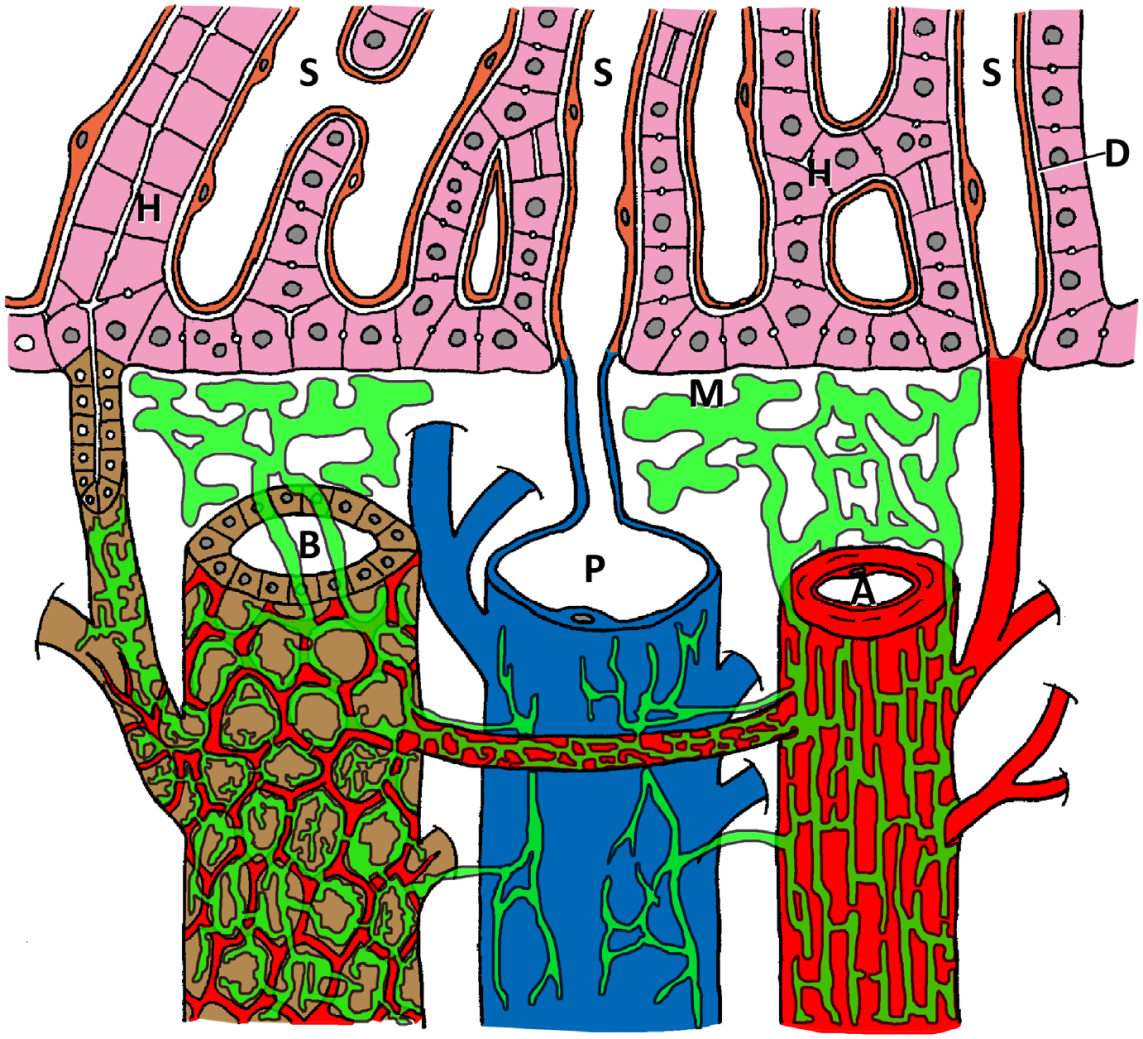
A schematic presentation of the human intrahepatic lymphatic system based on the current study. Lymphatic vessels, interlobular artery (A), bile duct (B), vein (P), hepatic cords (H), and sinusoidal capillaries (S) are colored green, red, brown, blue, pink, and orange, respectively. Lymphatic capillaries start from Mall's space and develop peculiar networks around the interlobular artery and bile duct and are connected by the lymphatics entwined around the peribiliary arterial plexus. The interlobular vein has a poor lymphatic distribution. D, Disse's space; M, Mall's space.

The intrahepatic periportal lymphatic system, as revealed in this study, is schematically illustrated in Figure 4.

### 3.4 Structure of lymphatic vessels at hepatic hilus

**FIGURE 5:**
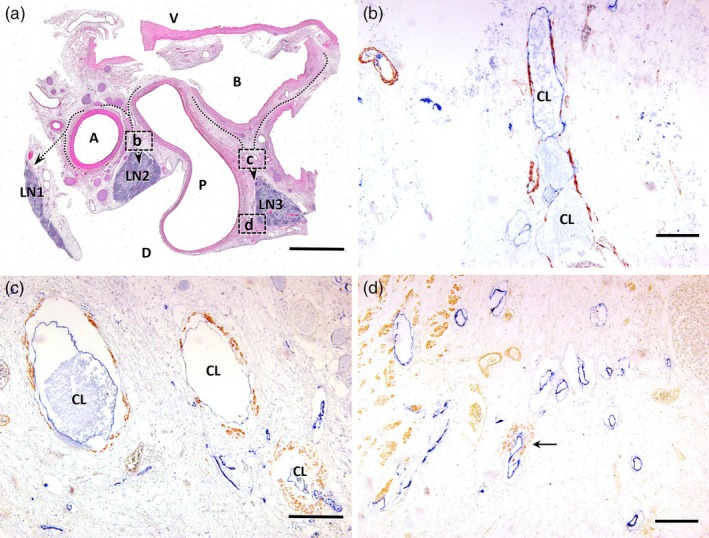
Light microscopic images of hematoxylin and eosin staining (a) and immunostaining (b) for podoplanin (blue) and α‐smooth muscle actin (brown) on adjacent horizontal tissue sections of the human hepatic hilus. (a) Cranial side view. Broken arrows indicate the running courses of the lymphatics from the external media of the right hepatic artery (A) and the bifurcation of the hepatic duct (B) to the dorsal lymph nodes (LN). LN1, 2, and 3 indicate lymph nodes at the hepatic hilus. The left hepatic artery is on the left side out of this image. D, dorsal side; P, portal vein; V, ventral side. (b) Higher magnification of an adjacent sectional image of the boxed area (b) indicated in (a). The collecting lymphatic vessels (CL) develop smooth media (brown) in the area between the right hepatic artery and portal vein. They decrease podoplanin immunoreactivity in proportion to their being CL. (c) Higher magnification of an adjacent sectional image of the boxed area (c) indicated in (a). The CL with smooth muscle media is seen between the hepatic duct and portal vein. (d) Higher magnification of an adjacent sectional image of the boxed area (d) indicated in (a). Numerous lymphatic capillaries immunopositive for podoplanin and a few CL (arrow) with smooth muscle media are seen in the dorsal area of the portal vein. Bars: 5 mm (a); 200 μm (b–d).

In the human hepatic hilum, anatomical structures demonstrating the transition from the intrahepatic to extrahepatic lymphatic system were identified. Horizontal sections of the hepatoduodenal ligament stained using HE revealed lymph nodes (LN1, LN2, LN3) located dorsally to the right hepatic artery, the confluence of the left and right hepatic ducts, and the portal vein (Figure 5a). On the ventral side of LN2, situated between the right hepatic artery and the portal vein, a collecting lymphatic vessel with a well‐developed smooth muscle layer was observed. Furthermore, the collecting lymphatic vessel exhibited reduced podoplanin immunoreactivity (Figure 5b). Similar to LN2, collecting lymphatic vessels with a prominent smooth muscle layer were observed in LN3, which is located dorsal to the confluence of the hepatic ducts and ventral to the portal vein. The hepatic hilar lymphatic capillaries, continuous with intrahepatic lymphatic vessels, formed collecting lymphatic vessels with smooth muscle layers as afferent lymphatic vessels to LN3 located dorsally in the hepatic hilum (Figure 5c). Additionally, lymphatic capillary development was predominant  in the dorsal region of the portal vein,  where fewer collecting lymphatic vessels observed (Figure 5d).

## 4 DISCUSSION

This study presents the first detailed visualization of lymphatic pathways surrounding the hepatic Glisson's capsule, revealing distinct lymphatic structures and demonstrating lymphocyte transport through CCR7/CCL21 signaling along the extravascular pathway.

Using the whole‐mount immunostaining method, lymphatic vessels with blind‐ended structures, presumed to be the origin of visible lymphatic vessels, were confirmed in the peripheral HT. The blind ends of the lymphatic vessels, where lymphatic endothelial cells are loosely arranged, are highly permeable and serve as gateways for the transport of fluid and lymphocytes.^13^, ^14^ Similarly, our previously published study on the hepatic venous lymphatic network confirmed the presence of blind‐ended lymphatic vessels on the walls of the peripheral hepatic veins.^12^ We previously reported that lymphatic fluid migrating through the extravascular pathway from the central vein enters these lymphatic vessels through their blind ends, suggesting that blind‐ended lymphatic vessels are the origin of mature lymphatic vessels within the liver.^12^ Given that this study also confirms the presence of blind‐ended lymphatic vessels around the peripheral HT, it indicates that mature lymphatic vessels originate in the peripheral HT tissues. This suggests that there are pathways for lymphatic fluid to flow from the peripheral parts of the liver to the hepatic hilum through the surrounding HT tissues. Therefore, these findings are in agreement with previous animal studies that identified the pathways for intrahepatic lymphatic fluid drainage through the periportal lymphatic system.^5^, ^15^ Moreover, in the intrahepatic HT, lymphatic vessels developed uniquely around the hepatic artery, portal vein, and bile duct. Notably, similar to the lymphatic networks in the digestive tract as shown by Shimoda et al., the lymphatic vessels around the bile ducts exhibited a characteristic tubulo‐saccular morphology.^16^ The tubulo‐saccular lymphatic structures observed around the bile ducts in the HT are presumed to be related to intrahepatic immune functions, similar to the defensive functions of the digestive tract, where numerous intestinal bacteria are present, and mucosa‐associated lymphoid tissues play a crucial role in immune function.^17^ Given that the bile ducts are contiguous with the digestive tract, they are a potential route of retrograde infection via the duodenal papillary region. The presence of a lymphatic network around the bile ducts in the liver is similar to that of the digestive tract and is an interesting finding. The distribution of intrahepatic lymphatic vessels and their continuity with extrahepatic lymphatic vessels represents a critically important area of study. In this study, the connection and lymphatic flow between intrahepatic and extrahepatic lymphatic vessels in the hepatoduodenal ligament at the hepatic hilum were investigated. Histological examination of the extrahepatic lymphatic vessels at the hepatic hilum identified the presence of lymphatic capillaries, collecting lymphatic vessels, and lymph nodes around the hepatic ducts, hepatic arteries, and portal veins. Although a comprehensive study of the structure and distribution of extrahepatic lymphatic vessels within the hepatoduodenal ligament was not conducted, we hypothesize that intrahepatic lymphatic vessels gradually increase in density toward the hepatic hilum and converge at the extrahepatic lymph nodes within the hepatoduodenal ligament. Furthermore, as suggested by Morine et al.,^4^ the lymphatic vessels within the hepatoduodenal ligament bifurcate into a lymphatic pathway toward the abdominal aorta and another pathway along the lesser curvature of the stomach extending to the posterior mediastinum.

Regarding the direction of lymphatic flow in the periportal lymphatic system, lymphatic fluid is presumed to flow predominantly in a centrifugal direction from the liver to the hepatoduodenal ligament. This assumption aligns with the general principle of lymphatic flow in which lymph flows from peripheral lymphatic capillaries into collecting lymphatic vessels, subsequently entering lymph nodes through afferent lymphatic vessels and exiting via efferent lymphatic vessels.^18^ Although the possibility of lymphatic flow toward the liver within the hepatoduodenal ligament cannot be entirely excluded, this study found limited evidence to support its presence. Additionally, previous reports have predominantly documented centrifugal lymphatic flow, referring to the direction from the liver toward the central lymphatic system.^4^, ^5^ Therefore, centripetal lymphatic flow directed toward the liver was unlikely. It is worth considering that the liver possesses lymphoid organ characteristics during the fetal stage given its role as a hematopoietic organ.^19^, ^20^ Consequently, centripetal lymphatic flow within the hepatoduodenal ligament may have been present during the fetal period. However, this study was unable to definitively identify the direction of lymphatic flow within the periportal lymphatic system.

In this study, India ink, which is a commonly used dye for lymphatic vessel research, was used.^21^, ^22^ Dynamics experiments using ink as a tracer for lymphatic fluid components strongly suggested that intrahepatic lymphatic fluid components enter Glisson's capsule from the sinusoids and permeate into Glisson's capsule lymphatic vessels through the extravascular pathway.^5^ We previously reported the presence of the extravascular pathway in the perivascular stroma, which runs from the central vein to the peripheral portion of the hepatic veins, serving as a transport route for lymphatic fluid.^12^ Extravascular pathways are networks for transporting nutrients and metabolic products. Collagen, particularly reticular fibers (Type III collagen), plays a significant role in this transport process.^23^ Reticular fibers, produced by reticular cells, form a network structure in organs, including the lymph nodes, spleen, thymus, and liver. They serve as the framework for organs and play crucial roles in cell transport, immune function, and homeostasis.^24^ Our study showed that the extravascular pathway is crucial for transporting lymphatic fluid from Glisson's capsule to capillary lymphatic vessels, similar to the hepatic venous lymphatic network. Previous studies using horseradish peroxidase (HRP) as a tracer for intrahepatic lymph dynamics have reported similar findings. Ohtani et al. demonstrated that HRP injected into the systemic circulation of rats flows through the sinusoids and Disse's and Mall's spaces into the periportal lymphatic system in the liver.^10^ Our results, consistent with those of Ohtani et al., importantly revealed that extravascular pathways play a critical role in the transport of intrahepatic lymphatic fluid to the lymphatic vessels in humans.

Additionally, in this study, the outflow pathways of the lymphatic fluid and lymphocyte components were examined. Our findings indicate that lymphocyte migration in the HT was highly associated with both CCL21/CCR7 signaling and CCL21 concentration gradients. Lymphocyte migration from the vascular system to the lymphatic system involves high endothelial venules (HEVs) in the cortex of lymph nodes and Peyer's patches.^25^ Chemokines, a group of molecules, play a crucial role in this migration. CCR7 is expressed in T, B, and mature dendritic cells, whereas CCL21, the ligand for CCR7, is expressed in HEVs. The signaling between CCR7 and CCL21 enables lymphocytes to migrate from the vascular system to the lymphatic system via HEVs.^26^, ^27^ Chemokine concentration gradients are involved in lymphocyte migration. Weber et al. demonstrated that CCL21 is present in the lymphatic endothelial cells of mouse skin, forming a gradient with a sharp decrease around the lymphatic vessels, thereby establishing hypotaxis in which lymphocytes migrate along the chemokine concentration gradient.^28^ In this study, CCL21 was expressed in reticular fibers, presumed to be a fluid pathway for lymphatic fluid, with high expression around lymphatic vessels in Glisson's capsule. Moreover, CCR7‐positive lymphocytes were present in these regions. Our study showed that lymphocytes entering Glisson's capsule are guided by CCR7/CCL21 signaling within the reticular fibers around the lymphatic vessels and transported into the lymphatic vessels of Glisson's capsule via the CCL21 concentration gradient. These findings indicate the existence of a lymphocyte transport system utilizing CCR7/CCL21 signaling and concentration gradients along with the permeation of lymphatic fluid into the lymphatic vessels through the extravascular pathway in the hepatic Glisson's capsule. Hepatocellular carcinoma and intrahepatic cholangiocarcinoma, representative primary malignant tumors of the liver, often metastasize to lymph nodes around the hepatic hilum. Metastasis of these lymph nodes is a significant prognostic factor, and the prognosis remains poor even after surgical intervention.^2^, ^3^ However, the mechanisms underlying this malignant behavior are poorly understood. It is generally believed that malignant tumor cells metastasize to the hepatic hilum lymph nodes via the intrahepatic periportal lymphatic system; however, detailed studies on this process are scarce.^5^ Our findings in the current study strongly support this hypothesis. However, we could not explain why lymph node metastasis through this pathway is associated with such a malignant course. Tumor cell migration and metastasis share many similarities with leukocyte transport, which is critically regulated by chemokines and their receptors. Chemokines and their receptors are crucial for regulating the tumor microenvironment, facilitating tumor growth, cell migration and invasion, angiogenesis, and metastasis.^29^, ^30^ Several studies have shown that specific chemokines expressed selectively in certain tissues attract specific CCR‐expressing tumor cells, thereby promoting metastasis. For example, in breast cancer, CCR7 is highly expressed in tumor cells and metastatic sites, and CCR7/CCL21 signaling is associated with lymph node metastasis and tumor progression.^31^ Similarly, oral squamous cell carcinoma expresses CCR7, and CCL21‐mediated signaling leads to lymphatic metastasis, correlating with poor prognosis.^32^ Furthermore, in tissue sections of liver tumors from patients with colorectal cancer liver metastases, the high expression of both CCR7 and CCL21 suggests that CCR7/CCL21 signaling promotes colorectal cancer liver metastasis.^33^ The CCR7/CCL21 transport system involved in intrahepatic lymphocyte transport, as elucidated in our study, may be strongly associated with lymphatic and intrahepatic metastasis of malignant liver tumors. Additionally, this transport system could play a role in the malignant dynamics of lymph node metastasis in intrahepatic malignant tumors.

Our study has some limitations that should be addressed in future research. First, the number of cadavers used in this study was limited. Pathophysiological changes in the liver, such as chronic hepatitis, liver cirrhosis, and metabolic‐associated steatohepatitis, generally induce structural alterations in the sinusoids and Glisson's capsule, which can profoundly affect lymphatic flow within intrahepatic lymphatic vessels. Moreover, clinical manifestations, including ascites in patients with liver cirrhosis and refractory ascites following liver resection in cases of severe hepatic fibrosis, could be significant contributors to the disruptions in lymphatic flow. Therefore, further studies involving a larger number of cadaveric livers are required to elucidate the details of the periportal lymphatic system. Second, lymphatic vessels might have been damaged during the preparation of whole‐mount liver specimens. Therefore, some parts of the lymphatic anatomy may have been inadequately elucidated. Third, there are limitations to the discussion of the directionality of lymph flow based on findings from human cadaver studies. Therefore, in this study, we examined both immune cell and lymphatic fluid components. Future studies on intrahepatic lymphatic flow in living humans using tracers, such as indocyanine green, are necessary to support our hypothesis.

## 5 CONCLUSIONS

Using human cadavers, we elucidated the intrahepatic periportal lymphatic system. The intrahepatic lymphatic fluid was found to enter the capillary lymphatic vessels within Glisson's capsule via the extravascular pathway. Additionally, lymphocyte components flow into these capillary lymphatic vessels through the CCR7/CCL21 transport system within the extravascular pathway. Furthermore, lymphatic components proliferate through visible lymphatic vessels originating from the peripheral HT, progressing toward the central HT, and ultimately reaching the hepatic hilum. This study is the first to clarify the intrahepatic periportal lymphatic system in humans.

## AUTHOR CONTRIBUTIONS

Yuto Mitsuhashi conducted the primary research and drafted the manuscript. Hiroshi Shimoda provided overall guidance on research planning and execution. Kotaro Umemura assisted in specimen dissection and organ removal. Takuji Kagiya and Kentaro Sato provided advice and support during the research execution, while Hirokazu Narita and Tomohiro Chiba contributed to research activities. Keinosuke Ishido supervised the research execution and manuscript preparation. Norihisa Kimura critically revised the article for significant intellectual content. Kenichi Hakamada, the corresponding author, supervised the research and manuscript preparation.

## FUNDING INFORMATION

This research did not receive any specific grants from funding agencies in the public, commercial, or not‐for‐profit sectors.

## 6 CONFLICT OF INTEREST STATEMENT

Authors declare no conflict of interest for this article.

## Data Availability

Authors declare no conflict of interest for this article.
